# Supplementary figures and images for: S-glutathionylation proteome profiling reveals a crucial role of a thioredoxin-like protein in interspecies competition and cariogenecity of Streptococcus mutans
Source: PLoS Pathog. 2020 Jul 27;16(7):e1008774. doi: 10.1371/journal.ppat.1008774 (PMC7410335; doi:10.1371/journal.ppat.1008774)

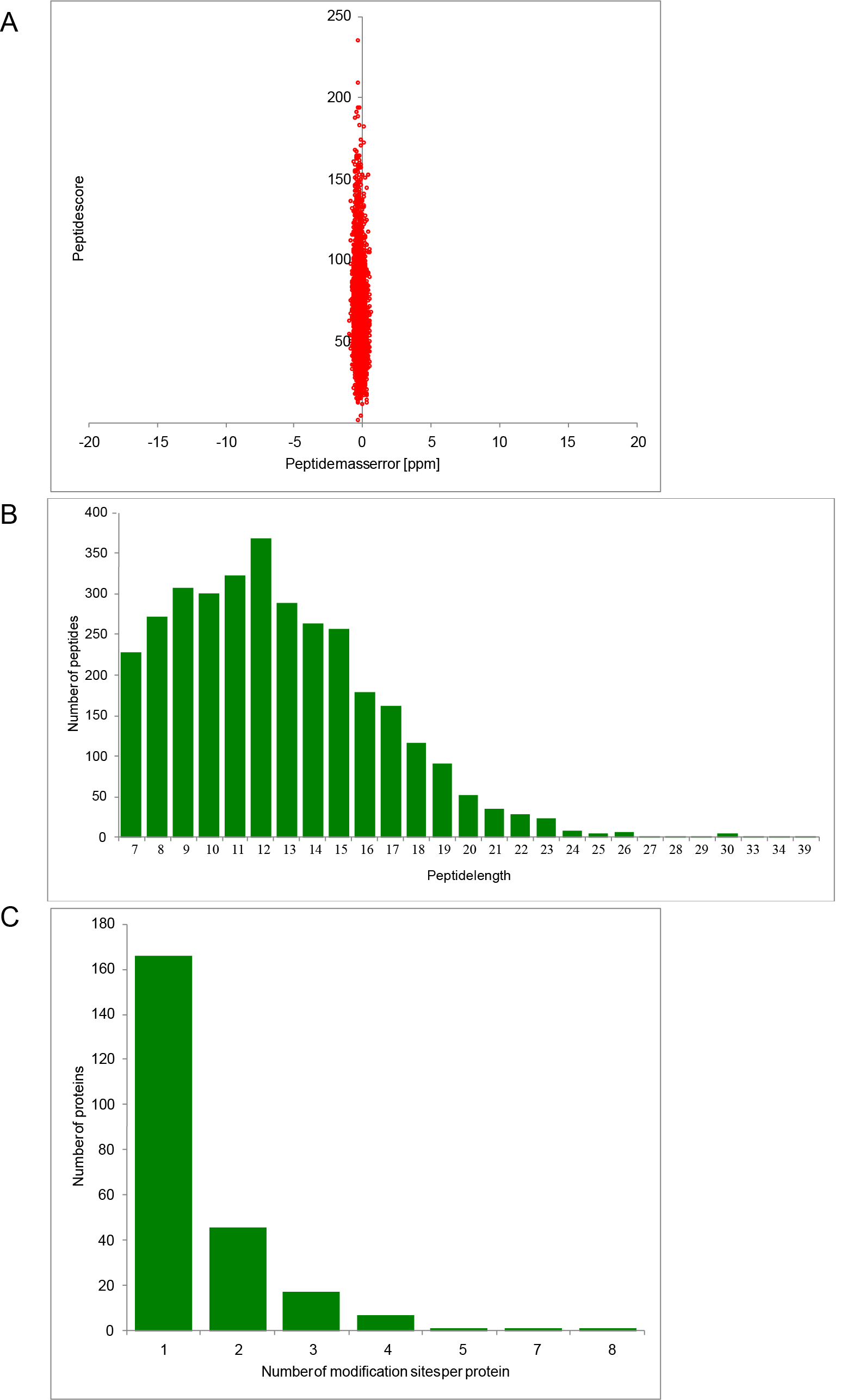

Supplement: S1 Fig — (A) Mass error distribution of all identified S-glutathionylated peptides in S. mutans UA159; (B) S-glutathionylated peptide length distribution in S. mutans UA159; (C) The number of S-glutathionylated sites identified per protein in S. mutans UA159. (TIF) [file ppat.1008774.s001.tif]

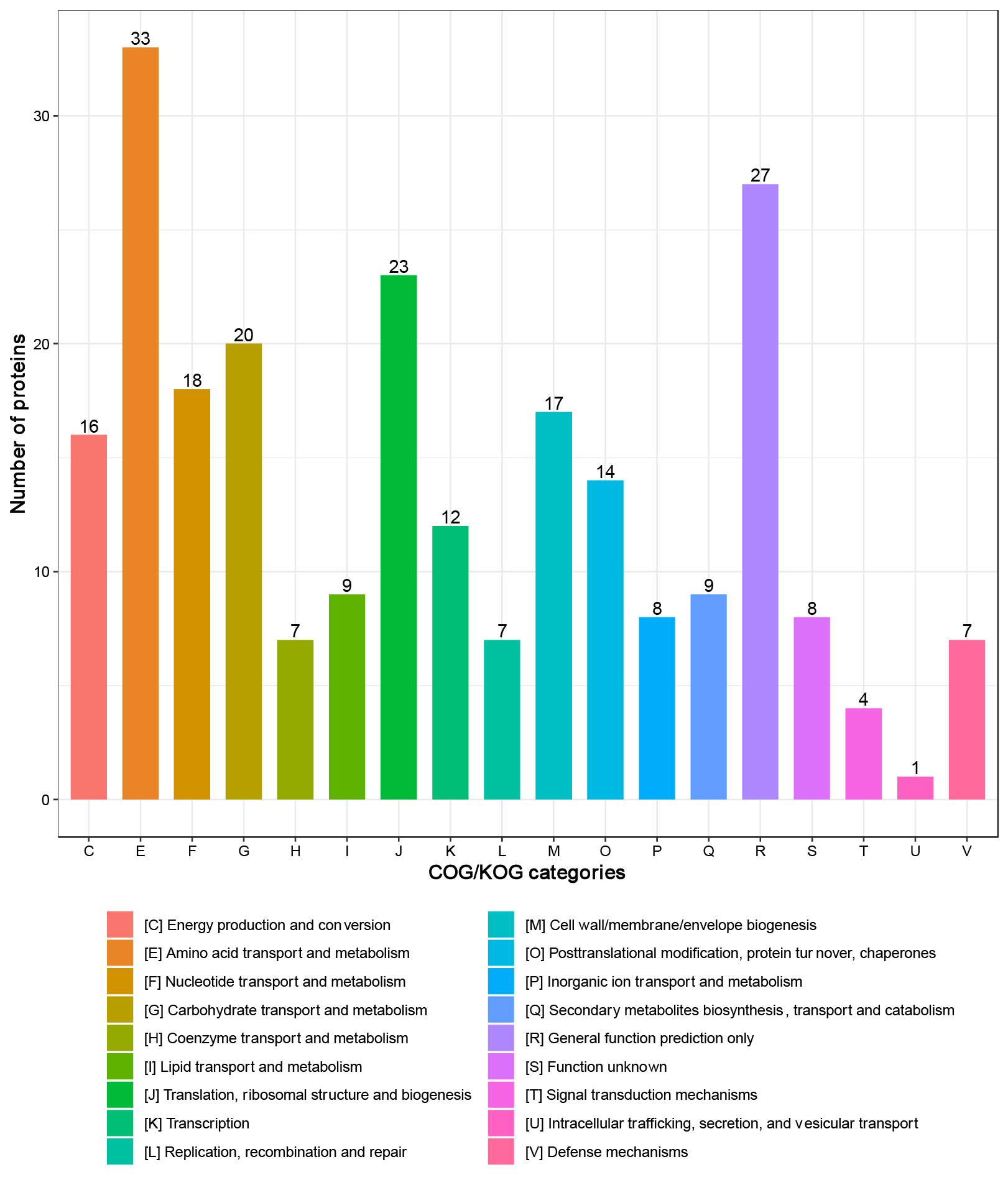

Supplement: S2 Fig — The numbers of modified proteins are labeled at the top of per category, below the histogram chart are the description of the categories. (TIF) [file ppat.1008774.s002.tif]

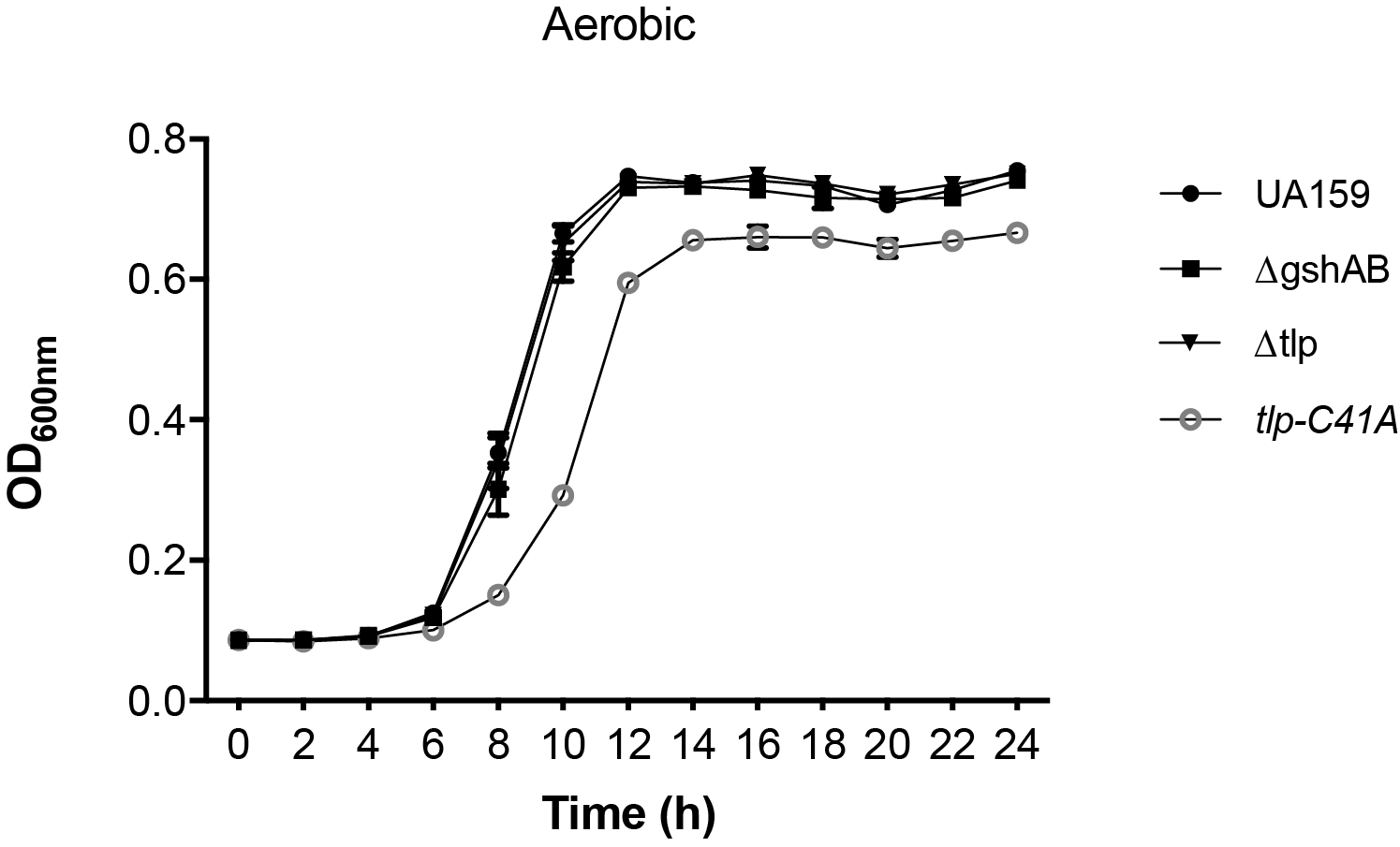

Supplement: S3 Fig — The results were averaged from 3 independent experiment and are presented as men ± SD. We didn't draw the error bars those are shorter than the height of the symbol. (TIF) [file ppat.1008774.s003.tif]
