# Supplementary material for: S-glutathionylation proteome profiling reveals a crucial role of a thioredoxin-like protein in interspecies competition and cariogenecity of Streptococcus mutans
Source: PLoS Pathog. 2020 Jul 27;16(7):e1008774. doi: 10.1371/journal.ppat.1008774 (PMC7410335; doi:10.1371/journal.ppat.1008774)
Supplement: Attachment — Submitted filename: Responses to reviewers-0615.docx [file ppat.1008774.s013.docx]

Responses to reviewers

**Summary**

Firstly, we sincerely send our appreciation to your careful reading and giving so many valuable comments and advice on the manuscript in the midst of pressing affairs and feel greatly honored by the recognition of our work! We have gone through this edition very carefully and explained the concerns as follows.

The Tlp null mutant has been constructed, and relevant data has been appended. We also purified the Tlp and its homolog protein of *S. pneumoniae* R6 (**Fig 5C**, protein accession: AAL00156.1, Bacteriocin transport accessory protein, which also contains a thioredoxin-like fold, but the antioxidative functions have not been studied), subsequent western blotting showed both these proteins could be glutathionylated at the conserved cysteine sites.

We have checked and improved our statistical analyses of the phenotype data. For better showing important and interesting proteins, we presented the significantly differentially modified proteins in a heatmap. Some explanation of the data has been rearranged, and additional information has been added to make it clearer.

**Significant Issues: Key Experiments Required for Acceptance**

**Reviewer #1: 1. The authors delete gshAB (which is responsible for synthesizing GSH) and Gst (which conjugates GSH to proteins) and determine the number of proteins having STM by the same methods. Surprisingly, 337 modified sites on 224 proteins were quantifiable. In some instances, levels of glutathionylation were increased for some of the STM proteins in these mutants. This indicates that there is another glutathionylation process occurring within the bacterium. This is very pertinent but was not discussed.**

Reply: We thank the reviewer for reminding us to discuss this vital point. We have discussed this point in the result part (line: 439-446). Briefly, we agree with the reviewer that there is another glutathionylation process within *S. mutans*, but not have been studied yet. Combining with other studies, we conclude that the non-enzymatic spontaneous glutathionylation process may be induced and even plays a predominant role after GST deletion.

**2. The homology of SMU_1788c to other known genes needs to be investigated thoroughly. There is a TlpA gene in S. pneumoniae that has been demonstrated to be necessary for survival against oxidative stress. Is this the same gene?**

Reply: We do appreciate the reviewer for indicating this vital flaw. We performed homology alignment with other known proteins in some common bacteria (Fig 5C). The results showed that these cysteine sites are substantially conserved. However, for TlpA (NP_358170.1) in *S. pneumoniae*, it performed low protein sequence identity (9.6%) with smu_1788c, indicating that they are unlikely to be the same gene.

**3. The authors should include results with a tlpA deletion mutant. In turn, describe how tlp-c141A compares to the null mutant? It is possible that by altering aa141 the activity of TlpA is altered in a fashion that makes in always activated – this would have a distinct phenotype than from its overall deletion. If a deletion mutant cannot be made, this should be indicated.**

Reply: We constructed the *tlp* null mutant strain successfully, subsequent assays indicated the *tlp* null mutant performed distinct phenotypes with C41A mutant, more concretely, *tlp* null mutant did not affect the oxidative resistance and competitiveness of *S. mutans* like C41A mutant. These results confirmed the reviewer’s opinion.

**4. There are broad concerns with regard to the statistics. These need to be addressed.**

Reply: We checked and corrected the statistic errors accordingly.

**Reviewer #2: The authors' approach to mapping all the S-glutathionylated proteins has been done nicely, and the data looks interesting. Nevertheless, the manuscript needs major revision. Some general concerns with the manuscript are as follows:**

Reply: We thank the reviewer for the positive appreciation of our work and for the time taken to review our manuscript. We have answered the remarks and concerns.

**1. the grammar is incorrect at many places.**

Reply: We checked and corrected the grammar errors throughout.

**2. citations of similar articles on S-glutathiolation from other bacteria are missing. Also, citations are missing from the result section, specifically, where the authors discuss about various proteins in functional classification (page 14-15).**

Reply: We have added these lost citations. (line 347:

[31] Kitten T, Munro CL, Michalek SM, Macrina FL. Genetic Characterization of a Streptococcus mutans LraI Family Operon and Role in Virulence. Infection and Immunity. 2000;68(8):4441-51. [32] Rolerson E, Swick A, Newlon L, Palmer C, Pan Y, Keeshan B, et al. The SloR/Dlg Metalloregulator Modulates Streptococcus mutans Virulence Gene Expression. Journal of Bacteriology. 2006;188(18):6718-. [32] )

**3. Growth condition mentioned in the materials and methods section is anaerobic. When authors talk about the growth profile of mutants, it is assumed that it was also carried out in the anaerobic chamber. If so, how do they expect that gshAB and gsT deletion would impact growth?**

Reply: We have applied the growth assay in aerobic conditions (in CO_2_ chamber). The results showed a similar growth profile with the anaerobic condition, indicating that *gshAB* and *gsT* deletion do not impact growth like C41A mutant do (**Fig S3**).

**4. It is also not clear that why gshAB and gsT mutants were chosen, when the bacteria was grown anerobically and all the thiolation work was carried out using added glutathione using lysates. Table S1 lists the fold change in the mutants and wt, while it is not clear that simialr peptides/thiolation-sites identified in both mutants and wt. Is it likely that the authors only picked common proteins/peptides identified in all three conditions. How do they justify excluding comparison with a redox-active agent with wt sample only rather than anaerobically grown wt and gshAB/T mutants for this work?**

Reply: We added the reason for choosing gshAB and gsT to manuscript (line: 428-433). Briefly, these two proteins are vital enzymes that participate in glutathionylation process. Thus, we think the deletion of them would impact glutathionylation of many proteins, and the differential modification might alter protein functions and further impact-related physiological processes, proteomics study could be helpful to reveal related mechanisms.

Table S1 also lists the MS intensity of each mutant (column M – O), and we did not deliberately choose common proteins/peptides identified in both mutants. In fact, all the modified peptides were listed in this table.

We agree with the redox-active agent might impact the glutathionylation levels of this bacteria, and this is valuable to be studied. In this study, we anticipated exploring the effects of these two genes on the protein glutathionylation, but oxygen has a critical impact on the cellular redox state and might also affect glutathionylation. Thus, we cultured these strains all under anaerobic conditions.

**5. Group AB and T descrription missing from the method section. it only becomes clear in later part of result.**

Reply: We described the group AB and T at the start of protein extraction step (line: 139), they were all treated by same way except the mass of label reagents.

**6. a lot of information from the result section about the function of various membrane proteins from the result section can go to discussion.**

Reply: We agree with the reviewer’s comment and removed most of the information in the result section about the function of various membrane proteins.

**7. It will be nice if the authors show some tables showing the relevant proteins related to their functional categories as table, and this is quite common for omics data.**

Reply: Table S7 listed all the functional categories of every protein. In addition, to highlight important metabolic pathway related proteins those are involved in the PPI network, we listed some proteins corresponding to the core nodes and their functions in **Table 1**.

**8. database search mentions search with UA159 database; however, Table S1 has protein accession numbers corresponding to GS5 too.**

Reply: Thanks for pointing out this mistake, we checked our experimental procedure and proteomics report, the database we searched against is *Streptococcus mutans* database, we have corrected it in our manuscript (line: 201).

**9. the data is descriptive except the tlp mutant, hence conclusions** **shoud be more suggestive rather than being close ended.**

Reply: Thanks for the suggestion, we now have altered the conclusions to more suggestive (line: 581-585).

**10.For the enrichment analysis of mutant mass spec data, it will be intersting to see a table in the text or a heat map of proteins rather than functional categories to highlight the important proteins affected in the mutants.**

Reply: We presented the significantly differentially modified proteins in a heatmap (Fig 4A).

**11. the rationale for calling Tlp a Trx like protein is missing. How does it compare with the known Trxs in terms of overall predicted structure, sequence similarity etc. Also, CXXC in Tlp does not have a positively charged amino acid next to cys and therefore thiol may not be a reactive one. The data related to Tlp with respect to caries is fine and it can be considered an important protein.**

Reply: To clarify the rationale for calling Tlp a Trx like protein, we now have rephrased this sentence “We identified a thioredoxin-like fold containing protein through **domain annotation** (Q8DSJ5, Table S7, named as Tlp in this study) coded by SMU_1788c, which contained modified Cys sites at its thioredoxin catalytic motif (C38PYC41) and C100 (Fig 5A and B, Table S1), moreover the structural prediction revealed a similar structure with classical Trx structure, which contains a highly conserved fold that is composed of five β-strands surrounded by four α-helices, and a CXXC catalytic motif.”

For Tlp, the motif sequence “GRSTC(38)PYC(41)QR^*^FAPKL” contains a positive aa Arginine adjacent to C41, it could cause the decrease of p*Ka* value.

**12. Discussion talks about the known methods to probe S-glutathionylation;** **however, relevant citation is missing. It would be more appropriate to compare the data with available data in literature.**

Reply: We added relevant citations (line: 614:

[23] Pan K-T, Chen Y-Y, Pu T-H, Chao Y-S, Yang C-Y, Bomgarden RD, et al. Mass spectrometry-based quantitative proteomics for dissecting multiplexed redox cysteine modifications in nitric oxide-protected cardiomyocyte under hypoxia. Antioxidants & redox signaling. 2014;20(9):1365-81.

[48] Filipovska A, Murphy MP. Measurement of Protein Glutathionylation. Current protocols in immunology. 2006;28(1)

[49] Gao X, Bedhomme M, Veyel D, Zaffagnini M, Lemaire SD. Methods for Analysis of Protein Glutathionylation and their Application to Photosynthetic Organisms. Molecular Plant. 2009;2(2):218-35.).

Comparing with the data in the literature is a great note, but we failed for various reasons. Different proteomics data were performed in different organisms, MS methods, statistical analysis methods, filter criterions and presenting form, and it is hard to find a universal criterion to compare different data.

**13. Figures are not readable.**

Reply: Sorry for our work mistake, we now have uploaded full resolution figures.

**14. String analysis shows the connection between identified proteins. Hence the conclusion that all the glutathionylated proteins interact is wrong. They are a part of a network.**

Reply: We corrected the conclusion accordingly (line: 404-405).

**15. Figure legend for 5B is not clear.**

Reply: We have rephrased this legend to make it clearer (Line: 862-864).

**16. A better title would be "S-glutathionylation proteome profiling reveals a crucial role of a thioredoxin-like protein in interspecies competition and cariogenecity of Streptococcus mutans".**

Reply: Thanks for the reviewer’s advice, we have altered our title as suggested.

**Reviewer #3: 1) I make a minor comment below that is related to describing the methodology in greater detail, and some of my comments here might be in part related to this. Having pieced together the methodology/samples used I have some technical issues that I think the authors should consider.** **What is used to discriminate potential artifacts/contamination that arose during sample preparation? I do not think that a** **negative control is described (non-treated).**

Reply: We thank the reviewer for the time taken to review our manuscript. For discrimination potential artifacts/contamination, we constructed rigorous criterions during the proteomics study of glutathionylation modification, including the use of alkylating agents (IAM and NEM) during protein extraction and the enzymatic reaction-based reduction of the S-glutathionylation modification. These methods are standardized and widely accepted. For details, please refer to the literature “Quantitative Profiling of Protein S-Glutathionylation Reveals Redox-Dependent Regulation of Macrophage Function During Nanoparticle-Induced Oxidative Stress” (PMID: 26700264)

**2) S. mutans cells are cultured in BHI in anaerobic conditions in this study. Is it not possible to perform the experiments in cells growing in various redox conditions? This would probably also help with studying changes in gshAB or gshT mutant backgrounds.**

Reply: We agree with the reviewer’s comment, and we reconstructed the growth curve under different aerobic condition, the result showed similar growth profile with anaerobic condition (Fig S3). For the competition assay, anaerobic condition is helpful for excluding the impact from oxygen, making the oxidants secreted by *S. sanguinis* and *S. gordonii* were the only resource of oxidative stress. Actually, the animal experiment is impossible performed under anaerobic, thus the result is convinced to prove the impact of gshAB and glutathionylation of tlp_C41 on the virulence of *S. mtuans.*

**3) Were replicate experiments carried out? I cannot see this described in the methods/results.**

Reply: Sorry for our carelessness, in the last version, this point was described in the figure legend. We performed at least 3 biological replicates for all the experiments and repeated the experiments at least 3 times except the proteomics study. We now have described this in the methods part.

**Part III – Minor Issues**

**Reviewer #1: 1. Using high-specific labeling with high sensitivity mass spectrometry the investigators identified what is claimed to be the entire S-glutathionylation proteome of S. mutans UA159. A total of 357 cysteine S-glutathionylation sites on 239 proteins were identified. How does the fact that 11.7% of the S. mutans proteome can be glutathionylated compare to** **E. coli and Aeromonas hydrophila (if not bacteria then in eukaryotes)? Is the percentage of S. mutans PTM proteins low or high in comparison to these? How is this process the same or different between the species this has been examined?**

Reply: We agree with the reviewer that this point needs to be clarified. We have deleted the describe of *E. coli* and *Aeromonas hydrophila* for avoiding misunderstanding, because there is no proteomics study about their protein glutathionylation. We now have compared this data with the data from the glutathionylation identification of Salmonella Typhimurium 14028 (0.68%, 38/55586) and Synechocystis sp. PCC6803 (5.12%, 383/7477) (line: 321 – 323). For Salmonella Typhimurium, this process is based on Top-down proteomics analysis. For Synechocystis sp., this process is based on biotin affinity chromatography.

**2. It would be quite helpful if the investigators provided the distribution of proteins having 1-4 glutathionylated sites (do most have 1 or more than 1?).**

Reply: The sites distribution data is shown in Fig S1C. And it showed 69.45% (166/239) of total PTM proteins having 1 site, for the proteins having 2, 3 and 4 modified sites, this data is 19.25% (46/239), 7.11% (17/239) and 2.93% (7/239), respectively.

**3. In the discussion the authors provide detailed explanation for why the approach taken is best. Yet the do not discuss the limitations of their approach, i.e. any possible reasons that the methodology employed may be undercounting the number of identified proteins that are subject to S-glutathionlyation. This should be added.**

Reply: Thanks for your suggestion, we discussed this in the manuscript (line: 616-623).

**Reviewer #2: Line 18: residues**

**Line 18: under conditions of oxidative...**

**Line 18: "at the condition of" can be changed to "during"**

Reply: Thanks for your careful review, we have now corrected these as you suggested.

**Line 25: iodoTMT...what does TMT stand for?**

Reply: ‘Tandem mass Tag’, we have added the full name at it first appeared.

**Line 27: Proteome profililing....(remove following)**

**Line 31: on interspecies competition...**

**Line 51: italicize S. mutans...follow this for whole manuscript.**

**Line 54: interspecies competition instead of competitiveness...**

**Line 61: addition of GSH, which causes an increase**

[Line 27~Line 61] Reply: Thanks for your careful review, we have now corrected these as you suggested.

**Line 69 to 72: ref for S. pneumonie, L. monocytogenes gsh work...**

Reply: We added the citations. (line: 72, 75

[6] Potter AJ, Trappetti C, Paton JC. Streptococcus pneumoniae uses glutathione to defend against oxidative stress and metal ion toxicity. Journal of bacteriology. 2012;194(22):6248-54.

[7] Reniere ML, Whiteley AT, Hamilton KL, John SM, Lauer P, Brennan RG, et al. Glutathione activates virulence gene expression of an intracellular pathogen. Nature. 2015;517(7533):170.)

**Line 89: hostile to what?**

Reply: For *S. mutans*, hostile to *S.sanguinis* and *S.gordonii.* We have corrected this in manuscript.

**Line 132: "The primers used in this listed are listed in Table S8." instead of performed.....**

Reply: Thanks for your careful review, we have now corrected these as you suggested.

**Line 196: is it MASCOT or Masquant?**

Reply: Maxquant, sorry for our carelessness.

**Line 203:** **iodo TMT 6 plex var statement not clear.**

Reply: “iodoTMT 6 plex var” is a parameter that needed to be set when searching database, it is the statement for isobaric label reagent, for our study, the modified cysteines were labeled after reduction, so “iodoTMT 6plex var” represents the glutathionylation sites.

**competition assay: ref for 50% BHI plate?**

Reply: We added the reference.

**Line 278: "reduced" to be changed as "reduction"**

Reply: Thanks for your careful review, we have now corrected it as you suggested.

**Line 284: segments or amino acids?**

Reply: segments, we have now corrected it in manuscript.

**Line 289: indispensable to be replaced by important...gshAB mutants are not lethal**

Reply: We have now corrected it as you suggested.

**Replace UNIPROT ids with SMU no. it is easy for teh readers to follow.**

Reply: We agree with your suggestion, we now have added the SMU no. to the protein ids.

**Line 310-311: which adherence assay? or what adhesin? citations missing for function of all the proteins discussed in page 15.**

Reply: We have added the citations.

**Page 18: ref for role of gshAB and gsT in S. mutans**

Reply: We have added the reference

.

**Reviewer #3: 1) It would be helpful if the method used to examine Cys-redox modifications was explained better/earlier in the manuscript. It took me a significant amount of reading (from other papers) to elucidate how it works and then determine caveats. A diagram, as part of a figure, could be especially helpful with this.**

Reply: Thanks for your suggestion, we appended a diagram for our proteomics study workflow.

**2) Line 468 “t****lp-C41A mutant strain were significantly decreased compared with UA159” – is this a significant contributing factor to the decreased virulence in vivo?**

Reply: We agree with your point, the growth inhibition of the bacteria could impact the virulence *in vivo*, and the growth inhibition is caused by C41A mutant, so, the glutathionylation of Tlp have important impact on the growth, subsequent decrease virulence.

**3) Which strain of UA159 is used? Recently it was found that certain strains of UA159 have a truncation in perR (Smu.593) that might impact how it responds to H2O2 etc.**

Reply: The full length perR strain obtained from Oral Microbiome Bank of China. To confirm this, we designed a pair of primers (Forward: ggctacttgatgatgctaggc, Reverse: tgactcacctcctatttccca) to amplify the whole sequence of s*mu.593* in our strain, subsequent sequencing indicated our sequence is totally identity with the sequence from GeneBank (SMU_RS02830). Last page shows the sequences of our sequencing result and the standard sequence.

**4) Is it known if gshAB is functional in UA159? I cannot find a careful study of GSH synthesis in S. mutans and a paper by Sherrill and Fahey suggests that synthesis is not possible (PMCID:** **PMC107044).**

Reply: We aligned the gshAB gene and protein sequence with other *S. mutant* strains but didn’t find a similar gene or protein in ATCC25715 and ATCC 33402 (the strains used in the mentioned study by reviewer), in addition, database searching by UniProt couldn’t find an annotated GSH synthase in these two strains. So, it seems like there is no GSH synthase in these two strains, they couldn’t synthesize GSH. For function of gshAB in UA159, there is only one study (PMID: 23872989) has explored it, showing that gshAB is important for oxidative resistance and competitive ability of UA159, in addition, we aligned its proteins sequence with the gshAB (WP_084828691.1) in *Streptococcus thermophilus*, they performed 71% sequence identities and same conserved catalytic domains. The glutathione synthesis function of gshAB in *Streptococcus thermophilus* has been proved in the study: “Production of glutathione using a bifunctional enzyme encoded by *gshF* from S*treptococcus thermophilus* expressed in Escherichia coli” PMID: 21683099.

**5) The quality of written English could be improved throughout.**

Reply: We have checked and corrected many grammar errors and rephrase some sentences through the manuscript.

**GeneBank Smu.593:** ATGGGACTGCATTCTCATAATAATGATCCGAATAAGGATGTTTATGATCATGTATTAACGCATTTAAAAGAAAAGCATATTCGTATTACTAAAACACGAGAAGCTGTTATTTCTTATATGATTAACTCCCGCCGTCATCCGAGTGCAGAACAAATTCATAAGGATCTTCTTCCTCAGTATCCAAGTATGAGTTTAGCAACGGTCTATAATAATTTGAAAGTCCTGATTGATGAAGGCTTTGTTGTTGAATTAAAGCTAAGCAATGATAATACAGCTTATTTTGATTTTATGGGGCATCAGCATTTGCATGTTGTTTGTGAGTCTTGTGGTAAGATTACTGATTTTGTAGACACTGATATTTCTCATATCAAGCAAGAAGCATATAAGCAGACAAATTATGTGATTACGAGAGCACAGCTTTTGATTTATGGTATTTGTCCAGACTGTCAAAGCAAGAAAACTAAAAAAGCCGATTAA
