# Supplementary material for: S-glutathionylation proteome profiling reveals a crucial role of a thioredoxin-like protein in interspecies competition and cariogenecity of Streptococcus mutans
Source: PLoS Pathog. 2020 Jul 27;16(7):e1008774. doi: 10.1371/journal.ppat.1008774 (PMC7410335; doi:10.1371/journal.ppat.1008774)
Supplement: Attachment — Submitted filename: Reponse to reviewers0630.docx [file ppat.1008774.s014.docx]

We do appreciate the reviewers for the positive appreciation of our work and for the time taken to review our manuscript. We have gone through this edition very carefully and addressed the issues as follows.

1. We are sorry for the language problem, to address this, we solicited opinions from professional language editors and revised them throughout the manuscript. The grammar and syntax issues have been addressed appropriately in this version.

2. All the figure files have been checked and modified to meet PLOS requirements by using PACE tool.

3. We submitted a supporting data table that contains all the underlying numerical data and statistical analysis for figure panels.
